# How the Ethylene Biosynthesis Pathway of Semi-Halophytes Is Modified with Prolonged Salinity Stress Occurrence?

**DOI:** 10.3390/ijms25094777

**Published:** 2024-04-27

**Authors:** Miron Gieniec, Zbigniew Miszalski, Piotr Rozpądek, Roman J. Jędrzejczyk, Małgorzata Czernicka, Michał Nosek

**Affiliations:** 1 W. Szafer Institute of Botany, Polish Academy of Sciences, Lubicz 46, 31-512 Kraków, Poland; m.gieniec@botany.pl (M.G.); z.miszalski@botany.pl (Z.M.); 2 Małopolska Centre of Biotechnology, Jagiellonian University, Gronostajowa 7a, 30-387 Kraków, Poland; piotr.rozpadek@uj.edu.pl (P.R.); roman.jedrzejczyk@uj.edu.pl (R.J.J.); 3 Department of Plant Biology and Biotechnology, Faculty of Biotechnology and Horticulture, University of Agriculture in Krakow, Al. Mickiewicza 21, 31-120 Kraków, Poland; malgorzata.czernicka@urk.edu.pl; 4 Institute of Biology and Earth Sciences, University of the National Education Commission, Krakow, Podchorążych 2, 30-084 Kraków, Poland

**Keywords:** 1-aminocyclopropane-1-carboxylic acid, common ice plant, comparative transcriptome analysis, elevated salinity, *Mesembryanthemum crystallinum*, new generation sequencing, RNA-seq

## Abstract

The mechanism of ethylene (ET)–regulated salinity stress response remains largely unexplained, especially for semi-halophytes and halophytes. Here, we present the results of the multifaceted analysis of the model semi-halophyte *Mesembryanthemum crystallinum* L. (common ice plant) ET biosynthesis pathway key components’ response to prolonged (14 days) salinity stress. Transcriptomic analysis revealed that the expression of 3280 ice plant genes was altered during 14-day long salinity (0.4 M NaCl) stress. A thorough analysis of differentially expressed genes (DEGs) showed that the expression of genes involved in ET biosynthesis and perception (ET receptors), the abscisic acid (ABA) catabolic process, and photosynthetic apparatus was significantly modified with prolonged stressor presence. To some point this result was supported with the expression analysis of the transcript amount (qPCR) of key ET biosynthesis pathway genes, namely *ACS6* (1-aminocyclopropane-1-carboxylate synthase) and *ACO1* (1-aminocyclopropane-1-carboxylate oxidase) orthologs. However, the pronounced circadian rhythm observed in the expression of both genes in unaffected (control) plants was distorted and an evident downregulation of both orthologs’ was induced with prolonged salinity stress. The UPLC-MS analysis of the ET biosynthesis pathway rate-limiting semi-product, namely of 1-aminocyclopropane-1-carboxylic acid (ACC) content, confirmed the results assessed with molecular tools. The circadian rhythm of the ACC production of NaCl-treated semi-halophytes remained largely unaffected by the prolonged salinity stress episode. We speculate that the obtained results represent an image of the steady state established over the past 14 days, while during the first hours of the salinity stress response, the view could be completely different.

## 1. Introduction

In their natural habitats, plants are continuously exposed to alternating stresses and among them, the occurrence of osmotic stresses; in particular, the impact of salinity stress has significantly increased in recent years. According to the data of the FAO Land and Plant Nutrition Management Service, soils affected by increased salinity currently constitute more than 6% of the total cultivated area (nearly 400 million hectares), and this share increases by about 1–2% annually. The harmful effect of increased salinity applies to about 30% of the irrigated lands devoted to major crop agriculture and it is responsible for a monetary loss of approx. 27.3 billion USD per year [1]. This phenomenon results mainly from deforestation, intensive fertilization, rainfall absence, and irrigation with low-purity water. The water used in irrigation contains on average up to 200–500 mg of soluble salts per litre and, according to Parihar et al. (2015) [2], a hectare of crops in many locations may receive from 3 to 5 tons of salt per year. The elevated salinity- or drought-induced osmotic stress can lead to the disruption of water management in plant cells and, as a consequence, cell shrinking or swelling can be observed [3]. Interestingly, the low water potential of high salinity-affected areas is often not the result of low water content, since these regions are often characterized by a moderate amount of precipitation [4]. In many places with salinity-affected soils, there is plenty of water; however, contamination with large amounts of soluble salts drastically decreases soil water potential and heavily disturbs water uptake. Plants exposed to salinity stress have to “cope” with two problems: besides limited water availability, they have to deal with high concentrations of ions, usually Na^+^ and Cl^−^, which can be toxic [5]. Despite the difficulties associated with water uptake and harmful ion concentrations, many plant species have developed mechanisms that allow vegetation in conditions of osmotic stress. Plants of areas naturally affected by high salinity, known as halophytes, have developed mechanisms allowing an undisturbed execution of the developmental programme under salinity stress. Halophytes’ tolerance to salinity stress mainly results from sophisticated osmoregulation, the accumulation of osmoregulatory substances, namely osmolytes, which allow the stabilization of cell structures when exposed to elevated salinity without interfering with the central metabolism of the cell. Osmoregulatory substances like glutamic acid and betaines (derivatives of glycine, proline, γ-aminobutyric acid, and δ-amino valeric acid) can be synthesized or taken from the environment. Such mechanisms are not working in the so-called glycophytes (literally sweet plants), which are susceptible to salinity stress, and even a slight increase in ion concentration may disrupt their developmental programme execution [6,7]. On the other hand, plants growing in dry areas, similarly affected by osmotic stress effects, can execute their developmental programme by employing specialized adaptations involving delay (desiccation postponement) and/or protection (desiccation tolerance) mechanisms against water loss. One of the strategies exploited by plants struggling with osmotic stress (drought or salinity) is also the implementation of CAM (Crassulacean acid metabolism) photosynthesis. CAM-performing plants fix carbon dioxide (CO_2_) during the night in the form of malate and then refix this in the light during the following day [8]. Nevertheless, all plants, C_3_ and CAM, are equipped with a Rubisco enzyme responsible for the final CO_2_ fixing [9]. High water use efficiency (WUE), and consequently, the limitation and delay of drying, makes CAM an attractive modification that seems to be an excellent remedy for the aforementioned environmental stressors. Some plants with the capability of shifting between C_3_ and CAM photosynthetic mode are valuable research objects. The daily rhythm of their photosynthetic activity is strongly dependent on their photochemical activity and the regulation of the production of many metabolites and, among others, also some phytohormones. It was shown that phytohormones like ABA can induce CAM metabolism [10,11,12].

Plants are continuously exposed to at least a few unfriendly environmental conditions, which are referred to as stressors. The response to both abiotic and biotic stressors requires well-tuned interaction which cannot be performed without phytohormone commitment. Evidence gathered throughout at least the last two decades indicates ethylene (ET) is an important modulator of plant response to both abiotic and biotic stresses [1,10,13]. This simple gaseous olefine has been recognized as one of the major plant hormones for almost a century [14,15,16]. Its production occurs in all known plant tissues and is often used in agricultural practice. Besides being used in response to a wide variety of stresses, ET is involved in a diverse array of plant growth and developmental processes, including germination, leaf and flower senescence, leaf abscission, cell elongation, fruit ripening, and nodulation [1,17]. The ET biosynthesis pathway is relatively simple: methionine is converted first to S-adenosylomethionine (SAM) and in the next step to 1-aminocyclopropane-1-carboxylic acid (ACC), the immediate precursor of ET and 5′-methylthioadenosine (MTA), and next, ACC is converted to ET, CO_2,_ and cyanide. Two main enzymes, ACC synthase (ACS) and ACC oxidase (ACO), are involved in this biosynthesis pathway. In past years, the role of CO_2_ as a fundamental regulator for the last step of ET biosynthesis was confirmed [18,19,20,21].

A vast number of experimental works confirmed a key modulator role for ET during the salinity response of many crops, e.g., grapevine [22], maize [23], and tomato [24]. In addition to the positive role, evidence confirming ET as the negative regulator in the plant–salinity stress interaction has also been provided [25]. Gharbi et al. (2017) [24] suggested that ET applied before salinity stress may support plant adaption to forthcoming disturbances, probably by stomatal conductance maintenance, improved water use efficiency (WUE), and osmotic adjustment. It is worth mentioning that ET-induced enhanced tolerance to salinity stress through the enhanced expression of enzymatic antioxidants was also suggested [26]. On the other hand, the inhibition of ET receptors in some plant species has been demonstrated. The authors indicated that it can be a factor in changed, decreased plant tolerance to salinity stress depending on the type of inhibited receptor or receptors [27,28,29,30]. In the ET biosynthesis pathway, one of the crucial and also rate-limiting enzymes is 1-aminocyclopropane-1-carboxylic acid (ACC) synthase (ACS). In some plant species under salinity stress, the level of *ACS* transcripts decreases dramatically. Also, the activity of another enzyme engaged in ET biosynthesis, ACC oxidase, and the level of *ACO* transcripts are mostly increased [31].

Most studies regarding ET involvement in salinity stress concern the response of crops, which are mostly glycophytes. In our opinion, while this approach is economically justified, it narrows the field of view since it concentrates on the mechanism occurring exclusively in plants susceptible to elevated salinity and the employment of halophytes and semi-halophytes brings an even more interesting background for such analysis. This approach allows insight into ET-dependent regulatory mechanisms in plants whose natural life cycle is inextricably connected with the presence of osmotic stress, either in the form of high salinity or drought. The main aim of our study was to determine how the key components of the ET biosynthetic pathway are modified by the ongoing presence of high salt concentrations in semi-halophytes. To achieve this, the employed semi-halophyte model, namely the common ice plant (*Mesembryanthemum crystallinum* L.), was subjected to multifaceted analysis on the 14th day of the salinity stress episode. *M. crystallinum* was also analysed as a plant resistant to different heavy metals [32,33,34]. Moreover, the common ice plant is a well-recognized CAM facultative plant model with an extremely high plasticity of photosynthetic apparatus. Recent reports confirmed that the common ice plant’s mesophyll cells rapidly (up to 24 h) undergo the process of functional CAM withdrawal, involving broad rearrangements of PSI (photosystem I) and PSII (photosystem II) protein expression, chloroplast ultrastructure, and photochemistry performance in response to salinity stress removal [35,36,37,38,39]. The features mentioned above of the plant model allow the study of another important aspect determining the regulation of ET biosynthesis, and consequently also affecting the ET regulatory system, parallel to changes in the intracellular concentration of CO_2_.

## 2. Results

### 2.1. Reflectance Parameters Results

**Figure 1:**
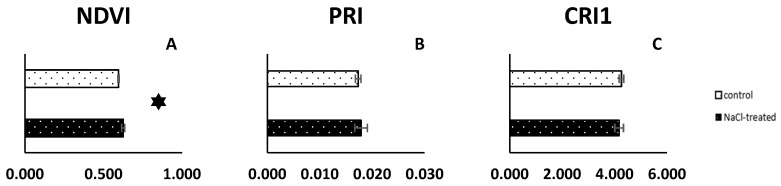
Reflectance parameters of control and 14 day NaCl-treated (+NaCl, CAM) *Mesembryanthemum crystallinum* L. plants. (**A**)—NDVI, normalized difference vegetation index; (**B**)—PRI, photochemical reflectance index; and (**C**)—CRI1, carotenoid reflectance index 1. Whiskers represent standard errors. The star indicate statistically significant differences between control and NaCl-treated plants (*N* = 4 for each experimental variant) according to the Student’s *t*-test. The absolute value for each parameter is given.

In the control C_3_ plants, the normalized difference vegetation index (NDVI) parameter value was significantly lower than in the NaCl-treated plants (0.59 and 0.63, respectively) (Figure 1A). No statistically significant differences in the photochemical reflectance index (PRI) and carotenoid reflectance index 1 (CRI1) parameters between control and NaCl-treated plants were observed on the 14th day of the experiment. PRI 0.017 and 0.018 and CRI 4.26 and 4.20 mean values were measured for control and NaCl-treated plants, respectively (Figure 1B,C).

### 2.2. Gaseous Exchange

**Figure 2:**
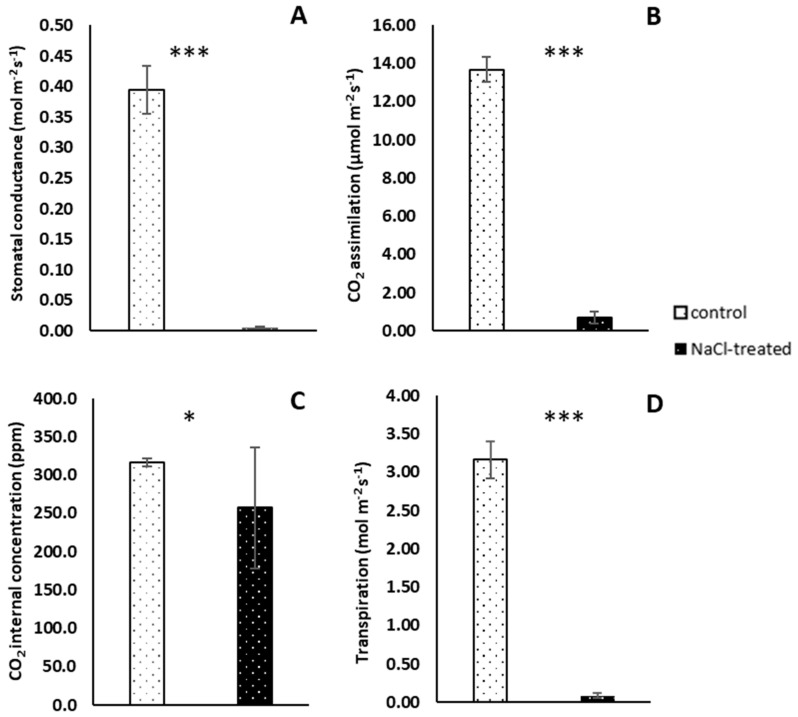
Gas exchange parameters of control and 14 day NaCl-treated (+NaCl, CAM) *Mesembryanthemum crystallinum* L. plants: (**A**)—stomatal conductance; (**B**)—carbon dioxide assimilation rate; (**C**)—carbon dioxide internal concentration; and (**D**)—transpiration. Whiskers represent standard errors. The stars above indicate statistically significant differences between control and NaCl-treated plants (*N* = 3 for each type plant) according to the Student’s *t*-test (* *p* ≤ 0.05, *** *p* ≤ 0.005).

Measurements of gas exchange parameters indicated that in the leaves of NaCl-treated plants, the photochemical activity was inhibited between 10.00 a.m. and 4.00 p.m. Whereas the value of stomatal conductance achieved in control plants was 0.394 mol m^−2^ s^−1^, in treated plants stomatal conductance was not observed (Figure 2A). Similar results in the case of transpiration rate (for control plants 3.16 mol m^−2^ s^−1^) and CO_2_ assimilation rate (13.69 and 0.72 µmol m^−2^ s^−1^ for control and treated plants, respectively) have been observed (Figure 2B,D). A higher concentration of internal CO_2_ in the leaves of control plants in comparison to treated plants has been demonstrated. However, in this case, an increased level of CO_2_ concentration (256.9 ppm) has been measured in NaCl-treated plants. This value is over 0.8 times lower than in control plants (Figure 2C).

### 2.3. Genome-Wide Identification of Expressed Genes in M. crystallinum under Salinity Stress

RNA sequencing of 16 cDNA libraries, i.e., eight biological replicates gained from the control (C_3_) and NaCl-treated (CAM) plants, was performed on the Illumina NovaSeq 6000 platform. For each sample over 6.3 Gbp total read bases and over 41 billion reads were generated (Supplementary Table S1). A higher number of clean reads was generated in the NaCl-treated samples (in total 332,922,540 clean reads and 50.3 Gbp, with an average of 6.3 Gbp per sample). For the C_3_ samples, 1% fewer clean reads were obtained (in total 329,964,332 clean reads and 49.9 Gbp, with an average of 6.3 Gbp per sample). The average GC content was approximately 46% both in CAM and C_3_. The clean reads from all samples were subjected to a de novo assembly of transcriptome resulting in 129,206 identified unigenes and 150,442 identified transcripts with an N50 of 1403 bp (Supplementary Table S2).

**Figure 3:**
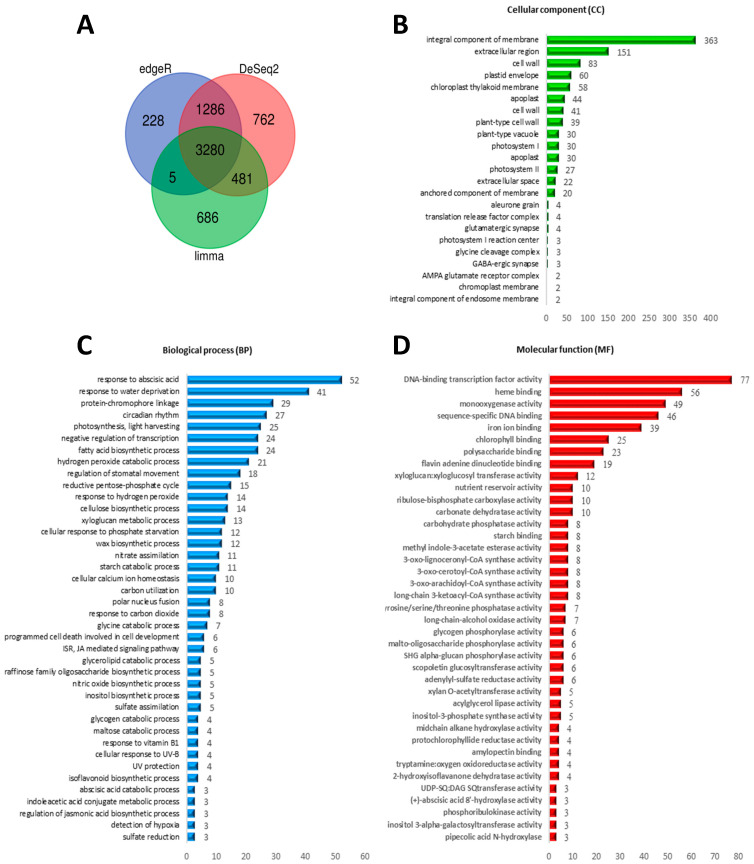
The expression patterns of genes in control and NaCl-treated (+NaCl, CAM) *Mesembryanthemum crystallinum* L. leaves. (**A**) Venn diagram showing common and specific differential gene expression levels identified using edgeR, DeSeq2 and Limma tools. (**B**–**D**) GO enrichment of DEGs in cellular component (CC) (**B**), biological process (BP) (**C**), and molecular function (**D**) categories, the x-axis indicates the number of genes and the y-axis indicates the GO terms.

Based on the read counts mapped to the set of assembled transcripts for each sample separately, an analysis of differential gene expression profiles between the two experimental groups was performed, using edgeR, DeSeq2, and Limma packages. The analysis of the Venn diagram enabled the identification of 3280 genes that were differentially expressed (DEGs—differently expressed genes) in all three tools used (Figure 3A). It was found that the direction of changes in the level of gene expression is the same regardless of the selected tool (Supplementary Figure S1, Supplementary Data S1). A total of 1918 (58%) genes were downregulated, while 1362 (42%) were upregulated in NaCl-treated when compared to control plants.

To better elucidate the biological functions of the DEGs in *M. crystallinum*, they were functionally annotated with gene ontology (GO) terms and classified as biological process (BP), molecular function (MF), and cellular component (CC). Among DEGs annotated with GO terms, 482 DEGs were assigned to 40 BP terms, 526 DEGs to 40 MF terms, and 1025 DEGs to 21 CC terms (Figure 3B–D, Supplementary Data S2).

Among DEGs assigned to 21 CC terms, the largest group was related to an integral component of membrane (GO:0016021), extracellular region (GO:0005576), cell wall (GO:0005618), plastid envelope (GO:0009526), and chloroplast thylakoid membrane (GO:0009535) (Figure 3B). Over 91% of DEGs were characterized by down-expression and these were genes related mainly to photosystem I, photosystem II, chloroplast thylakoid membrane, and envelope, as well as plant-type vacuole, aleuorone grain, and glycine cleavage complex (Table 1, Supplementary Data S2). 

The dominant BP and MF subcategories for which upregulated genes in NaCl-treated (CAM) plants were assigned were response to abscisic acid (GO:0009737), response to water deprivation (GO:0009414), fatty acid biosynthetic process (GO:0006633), nitric oxide biosynthetic process (GO:0006809), DNA-binding transcription factors activity (GO:0003700), and flavine adenine dinucleotide binding (GO:0050660), respectively (Figure 3C,D, Supplementary Data S2). For those downregulated in NaCl-treated plant genes, the dominant enriched BP and MF subcategories included circadian rhythm (GO:0007623), photosynthesis light harvesting (GO:0009765), hydrogen peroxide catabolic process (GO:0042744), reductive pentose-phosphate cycle (GO:0019253), starch catabolic process (GO:0005983) and chlorophyll-binding (GO:0020037), iron-binding (GO:0005506), monooxygenase activity (GO:0004497), polysaccharide binding (GO:0030247), and ribulose-bisphosphate carboxylase activity (GO:0016984), respectively. 

The functional analysis of DEGs under NaCl treatment allowed the identification of ET-related genes (ERGs) as well as those involved in the abscisic acid (ABA) catabolic process, nitric oxide biosynthetic process, regulation of stomatal movement, reductive pentose-phosphate cycle, glycine catabolic process, and also those involved in chlorophyll-binding, photosystem I, photosystem II, starch binding, and plant-type vacuole (Table 1).

Among the 3280 DEGs, we detected 15 ET-related genes (ERGs), of which 12 were upregulated in NaCl-treated plants including 1-aminocyclopropane-1-carboxylate oxidase (*ACO*), ethylene receptor (*ETR*), protein reversion-to-ethylene sensitivity 1 (*RTE1*), and ethylene-responsive transcription factors, i.e., *RAP2.3*, *RAP2.4*, *RAP2.12*, *ERF61*, *ERF80*, and *AP2LI* and WRKY transcription factor 23 (*WRKY23*) (Table 1, Supplementary Data S2). We found that the *RAV2* (AP2/ERF and B3 domain-containing transcription repressor RAV2) gene was downregulated in the NaCl-treated samples.

Most DEGs assigned to the abscisic acid (ABA) catabolic process (GO:0046345) accumulated higher transcript levels under salt stress, i.e., phosphoenolpyruvate carboxylase kinase 1 (*PPCK1*), protein C2-domain ABA-related 4 (*CAR4*), homeobox-leucine zipper protein ATHB-7 (*ATHB7*), homeobox protein BEL1 homolog (*BEL1* and *BELH1*), protein phosphatase 2C (*P2C24*, *P2C37*, and *P2C56*), fructose-bisphosphate aldolase 2 (*ALFC2*), aquaporin PIP2-2 (*PIP22*), protein early-responsive to dehydration 7 (*ERD7*), annexin D4 (*ANXD4*), galactinol synthase 2 (*GOLS2*), Ninja-family protein AFP2 (*AFP2*), primary amine oxidase (*AMO*), serine/threonine-protein kinases (*SAPK2*, *SRK2I*, and *CIPK1*, and *Y1141*), calcium-dependent protein kinases (*CRK*, CDPKO), membrane proteins (*RMR41*, *CRPM4*, *HHP1*, and *ECP44*), and ABA-related transcription factors (*AP2L1*, *SRM1*, *MYB88*, *MYBS3*, *MY102*, *NAP2*, and *NAC2*) (Table 1).

However, abscisic acid 8′-hydroxylase (*ABAH2*, *ABAH4*) and RNA-binding protein CP29B (*CP29B*) genes were downregulated under NaCl treatment.

Two genes involved in the nitric oxide biosynthetic process, i.e., encoded nitric oxide synthase (*NOS*) and primary amine oxidase (*AMO*), as well as 10 genes related to the regulation of stomatal movement, i.e., encoded beta carbonic anhydrase (*BCA1*, *BCA2,* and *CAH2*), protein phosphatase 2C (*P2C37* and *P2C56*), serine/threonine-protein kinase SRK2I (*SRK21*), protein zinc induced facilitator-like 1 (*ZIFL1*), potassium channel AKT1 (*AKT1*), sodium/hydrogen exchanger 2 (*NHX2*), and transcription factor MYB61, were overexpressed in NaCl-treated plants (Table 1).

Salt stress in *M. crystallinum* decreased the expression of genes related to the reductive pentose-phosphate cycle (Benson–Calvin cycle), photosynthesis, glycine catabolic process, and starch binding and localized in the vacuole. 

For the reductive pentose-phosphate cycle, it was possible to detect six DEGs encoding ribulose bisphosphate carboxylase small chain (*RBS1*, *RBS3*, *RBS4*, *RBS5,* and *RBS6*), three phosphoribulokinase (*KPPR*) genes, and Rubisco accumulation factor 1.2 (*RAF2*), glyceraldehyde-3-phosphate dehydrogenase GAPB (*G3PB*), and sedoheptulose-1,7-bisphosphatase (*S17P*) genes (Table 1).

The largest group of DEGs characterized by reduced expression levels under salt stress were genes related to photosynthesis, including genes related to chlorophyll-binding a-b (*CB2A*, *CB2D*, *CB4C*, *CB21*, *CB23*, and *CB27*), photosystem I reaction centre (*PSAD* and *PSAH*), and photosystem II, i.e., RNA-binding protein CP29B and oxygen-evolving enhancer protein 3-1 genes (Table 1). Moreover, a large group of downregulated genes were chloroplast-encoded genes.

Among the downregulated genes involved in the glycine catabolic process, there were mitochondrial genes such as glycine cleavage system H protein (*GCSH*), aminomethyltransferase (*GCST*), and glycine dehydrogenase (*GCSP*) (Table 1).

The expression of two genes related to starch binding, i.e., 4-alpha-glucanotransferase DPE2 and phosphoglucan phosphatase DSP4, was suppressed by NaCl treatment.

We observed negative changes in the expression of genes related to the vacuole, i.e., protein NRT1/ PTR family 8.3 (*PTR2*), vacuolar cation/proton exchanger 3 (*CAX3*), two-pore calcium channel protein 1 (*TPC1*), aquaporin (*TIP11* and *TIP21*), KDEL-tailed cysteine endopeptidase (*CEP1*), cyanidin 3-O-glucoside 5-O-glucosyltransferase (*AA5GT*), and transporters (ABC transporters, nitrate transporter 2.5, aluminium-activated malate transporters, sugar transporter ERD6-like, Na/Ca exchanger NCL, and organic cation/carnitine transporter 3) (Table 1). 

### 2.4. Gene Expression Analysis

**Figure 4:**
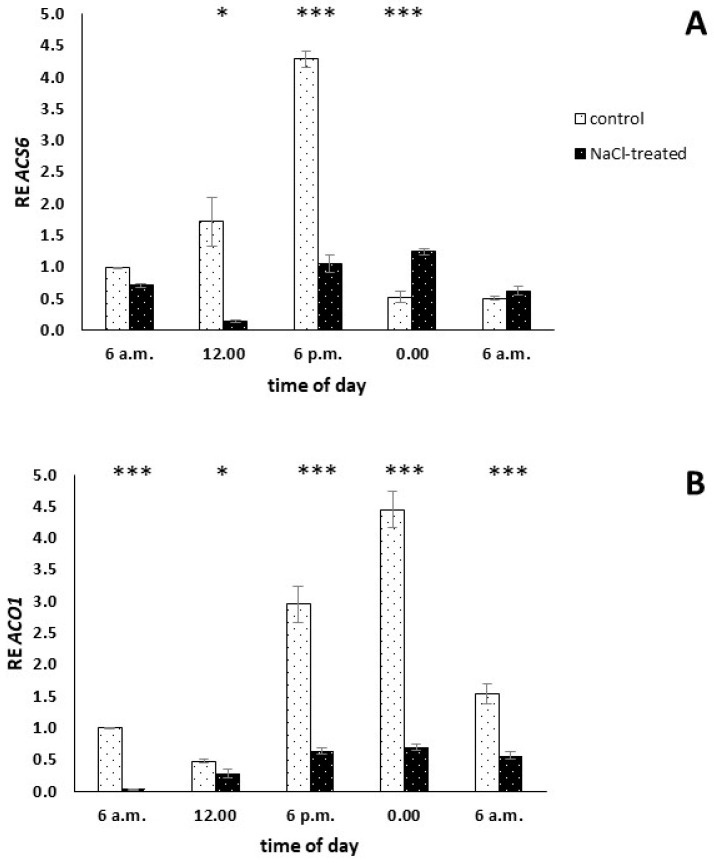
Expression of key genes involved in ethylene (ET) biosynthesis pathway, namely 1-aminocyclopropane-1-carboxylic acid (ACC) synthase 6 (*ACS6*) (**A**) and ACC oxidase 1 (*ACO1*) (**B**) orthologs analysed every 6 h during 24-h-long course in control and NaCl-treated (+NaCl, CAM) *Mesembryanthemum crystallinum* L. plants. NaCl treatment was applied for 14 days. Whiskers represent standard errors. The stars indicate statistically significant differences between control and NaCl-treated plants (*N* for *ACS6* = 3; *N* for *ACO1* = 4) at the specific time point according to the Student’s *t*-test (* *p* ≤ 0.05, *** p < 0.001).

To determine how salinity stress occurrence affects the expression profile of genes involved in the ET biosynthesis pathway, ACO and ACS, in leaves of *M. crystallinum* plants, quantitative PCR was employed. Our analyses showed that the daily courses of the transcript amounts of enzymes directly involved in ET biosynthesis, precisely ACS6 and ACO1, were disturbed by a salinity stress episode (Figure 4). A comparison of ACS6 daily expression in control (C_3_) and NaCl-treated (CAM) plants suggests at least some stress-induced modification. Our analyses revealed statistically significant differences in the expression level of genes between control and NaCl-treated plants in three out of five analysed time points (Figure 4A). Increased ACS6 expression was measured in C_3_ plants at 12:00, 6 p.m., and 00:00. In the case of ACO1, the salinity-induced modification was even more evident (Figure 4B). In salinity-stressed (+NaCl, CAM) plants, the expression of ACO1 was downregulated in all analysed time points in comparison to control plants. While in CAM plants ACO1 expression levels were more or less similar, in C_3_ plants the 24-h course of expression showed a clear differentiation between analysed time points with the highest expression reaching an over 6-fold increase in comparison to NaCl-treated plants at 00:00 a.m. (18 h past the start point). In control plants, the daily course of ACS6 and ACO1 expression was found to be similar; however, in the case of the latter gene, the increase in expression in a daily rhythm was delayed by 6 h.

Although products of ACS10 expression are not directly involved in the ET biosynthesis pathway we also estimated the daily course of this gene expression profile. A significant difference between control and salinity-stressed (CAM) plants was observed in only one out of six analysed time points, precisely at 12:00 (6 h past the beginning of the cycle) (Supplementary Figure S2). In this case, the salinity stress impact on the ACS10 daily expression course was rather scarce.

### 2.5. ACC Content Analysis

**Figure 5:**
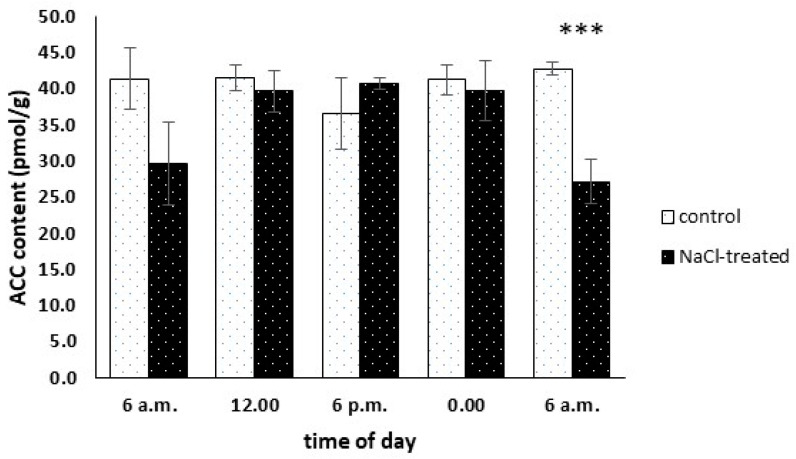
1-aminocyclopropane-1-carboxylic acid (ACC) content analysed every 6 h during the 24-h-long course in control and NaCl-treated (+NaCl, CAM) *Mesembryanthemum crystallinum* L. plants. NaCl treatment was applied for 14 days. Whiskers represent standard errors. The stars above indicate statistically significant differences between control and NaCl-treated plants (*N* = 6) at the specific time point according to the Student’s *t*-test (*** *p* ≤ 0.005).

1-aminocycloprapane-1-carboxylic acid (ACC) is the immediate precursor of ET in plants. ACC is synthesized by the ACS enzyme from S-adenosyl-L-methionine (SAM) and later converted to ET by the ACO enzyme. To determine how salinity stress occurrence affects the profile of ACC biosynthesis in M. crystallinum, a liquid chromatography-mass spectroscopy method was employed. In three out of five analysed time points, we found no statistically significant differences in ACC amount between control and salinity-treated plants. However, in stress-affected plants we found significantly lower amounts of ACC at 6.00 am (Figure 5). While in control plants the ACC concentration ranged between 36 and 43 pmol g^−1^ and reached its maximum after 24 h from the start point, in NaCl-treated (CAM) plants it ranged from 29 to 42 pmol g^−1^ with a maximum at 6 p.m. (Figure 5).

## 3. Discussion

To discuss the role of salinity on chosen phytohormones an evaluation of the general condition of the plants is necessary. NDVI, PRI, and CRI values are related to the level of plant stress. Reflectance measurements enable the estimation of part of the light reflected from the leaf surface. Higher values of NDVI (in the range from −1.0 to 1.0) indicate better plant condition. We also know that higher values for PRI and CRI parameters can be related to an elevated stress level in plants [40,41,42]. The analysis of reflectance parameters did not show significant discrepancies between control (C_3_) and NaCl-treated plants (CAM). Also, NDVI values between 0.5 and 1.0 indicated good conditions of not only control, but also NaCl-treated plants. No significant differences between PRI and CRI suggest that treated plants can cope with salinity stress and 14 days of NaCl irrigation do not influence photochemical apparatus efficiency. Nevertheless, gas exchange measurements proved CAM photosynthesis mode occurrence in plants treated for 14 days with NaCl solution. *M. crystallinum* CAM-performing plants exhibit a specific pattern of day/night CO_2_ assimilation different than that of C_3_. During the day, the stomata of CAM plants remain mostly closed and, as a consequence, the assimilation of external CO_2_ and transpiration is inhibited. The CO_2_ is absorbed during the night and fixed mainly by the cytoplasmic phosphoenolpyruvate carboxylase (PEPC) enzyme and later, during the day, carbon is successively used as a substrate in the Calvin–Benson–Bassham (CBB) cycle [43]. The obtained results are a confirmation of these processes in NaCl-treated leaves. Other than in the leaves of control plants, which realize C_3_ photosynthesis and assimilate CO_2_ during the day, transpiration and total CO_2_ assimilation in NaCl-treated plants was inhibited. Internal CO_2_ present in CAM plants has been fixed during the night and its level decreases as the photosynthesis process progresses during the day. Mentioned observations were also confirmed *via* transcriptome analysis. As we know, photosynthetic activity (CO_2_ fixation) in CAM plants is much lower compared to C_3_ plants [44,45]. Thus, we may expect that the expression of genes responsible for small subunits (nuclear-encoded) will be lower, as will also be the case for other enzymes taking part in the Calvin cycle. All identified genes of a small chain of D-ribulose 1,5-bisphosphate carboxylase/oxygenase (Rubisco), the main enzyme engaged in carbon dioxide absorption in C_3_ photosynthesis mode, are downregulated in treated plants. Also, a lower expression level of the chloroplastic *Rubisco accumulation factor 1.2* gene was noted. Rubisco is a bifunctional enzyme responsible not only for CO_2_ fixation. This enzyme also plays a crucial role in the photorespiration process (oxygen fixing in light conditions) whose product is, among others, glycine, a substrate for the synthesis of glutathione, which is a factor involved in a protected process under different plant stresses [46,47]. This process serves as an energy sink preventing the over-reduction of the photosynthetic electron transport chain, especially under stress conditions that lead to reduced rates of photosynthetic CO_2_ assimilation. We suppose that two-week salinity stress could induce this process in *M. crystallinum* plants. The downregulation of genes of enzymes involved in the glycine catabolic process can protect against glycine degradation and lower the abundance of this substrate. According to previous research [38,39], we know that 2-week salinity stress induction *via* NaCl solution leads to a change in photosynthetic type from C_3_ to CAM in *M. crystallinum* plants. This approach does not cause irreversible damage to our model plant machinery. Nevertheless, salt conditions also influence changes in metabolic machinery, signalling pathways, hormonal and gene expression regulation in halophyte plants [47]. Following lower photosynthetic activity, a lower amount of photosynthetic machinery in CAM plants is also expected. Thus, all genes encoding proteins necessary for *chla* and *chlb* binding in thylakoids in reaction centres and photosynthetic antennae are expressed in lower amounts. The same expectation can also explain lower reductase protochlorofilides, and proteins necessary for PSI (photosystem I) building. The inactivation of both PSII (photosystem II) and PSI (photosystem I) by increasing the cytosol’s NaCl levels has been described earlier [48,49].

In addition, genes responsible for NO synthesis like nitric oxide synthase are activated. Several metabolic processes can be controlled by this molecule, but the most important are the protection of plants against the undesirable effects of free radicals, the modulation of stress resistance gene expression, and finally, programmed cell death. Among different phytohormones, the gaseous olefin ethylene (ET), next to salicylic acid (SA), abscisic acid (AB), and jasmonic acid (JA), is usually mentioned when plant interactions with both biotic and abiotic environmental stressors are discussed [50,51,52]. We detected that over fifty genes involved in response to ABA have been activated. As ABA is one of the most important factors controlling stomatal aperture, we can suspect that a sensitive system which allows the control of excess transpiration is necessary in plants exposed to salinity. In addition to this, salinity stress in *M. crystallinum* induces CAM metabolism that is the opposite of the C_3_ plant stomata daily opening/closing rhythm. As shown in our experiments, this needs a higher expression of genes responsible for ABA. This process involves the binding of ABA to PYR/RCAR receptors [53,54]. Genes responsible for ABA catabolic processes are strongly lowered. Again, this would indicate that ABA-responsible mechanisms are necessary in plants exposed to salinity (CAM).

In our opinion, for all plant–environmental interactions in which ET is involved, it is particularly important to understand the contribution of this phytohormone in response to salinity stress. Experimental data obtained mostly from transgenic plant analyses show that salinity stress affects almost all components of both ET biosynthesis, as well as the signal transduction pathway. The increase in ET-responsive genes transcription factors and ET receptors together with the decrease in transcription repressors could point out the fact that plants exposed to salinity are better prepared to respond to different biotic and abiotic stresses. Thus, due to exposure to salinity, tested plants can be prepared for other stresses in a mechanism known as the cross-tolerance mechanism. In the context of expression level fluctuations of the gene involved in the ET biosynthesis and signalling pathway under salinity stress, *A. thaliana* seems to be the best characterized plant [1]. Based on the expression profile related to ET synthesis, perception, and action, we suppose that prolonged salinity stress influences ET activation. Nevertheless, this factor does not affect semi-halophyte *M. crystallinum* plants so strongly as to induce a burst of ET biosynthesis. We detected a higher level of 1-aminocyclopropane-1-carboxylate oxidase gene expression, a gene of the key enzyme responsible for the catalysis of ET biosynthesis from ACC substrate synthesis [55]. The increase in *ACO* transcript numbers was also observed in tobacco [29]. Short and long salinity stress induced the upregulation of genes engaged in ET biosynthesis in cotton (*Gossypium*) like *ETR*, *EIN*, and *ERF* [26]. Surprisingly, in our analysis the expression of the *ACO5* gene was decreased. A similar observation was made during the analysis of *Plantago major* transcriptomic profiling [56]. A higher level of expression in NaCl-treated plants was also described in the case of the ethylene response 1 (ETR-1) gene. ETR is a receptor localized in the endoplasmic reticulum and constitutes the negative regulator of the ET signalling pathway [56]. Nevertheless, *ETR* genes expression was inhibited under salinity stress in *A. thaliana*. Moreover, *A. thaliana* mutants with an *etr* loss-of-function were more tolerant to salinity stress. In our opinion, salinity treatment was not so intensive a stress factor as to induce the complete ET perception and signalling pathway, but genes of some factors responsible for plant response under stress conditions were activated. Among them, the most important are transcription factors (TFs) able to interact with other genes to activate or repress their transcription. The largest number of TFs belong to the ET response factors (ERFs) superfamily. In *M. crystallinum* NaCl-treated plants, some genes of TFs were upregulated. RAP2-3, RAP2-4, and RAP2-12 [57,58] are factors responsible for positively regulating low oxygen, oxidative, osmotic, and drought stress and most importantly, ET-mediated development. *RAP2-12* gene overexpression resulted in a lower concentration of H_2_O_2_ and increased accumulation of proline in *Arabidopsis* under salinity stress [59]. Similar to RAP, ERF61 and ERF80 play a crucial role in the stress response of plants [60]. On the contrary, the expression of *ERF53,* responsible for the positive regulation of response under heat stress, was lower in CAM plants, similar to the *AP2/ERF* gene, whose product also plays a crucial role in positive stress response. These findings can indicate that during the leaves material collection, plants were at the stage of adaptation and the development of proper mechanisms for salinity stress after a temporary increase in ET biosynthesis during NaCl-treatment. Our theory is supported by the downregulation of *RTE-1* gene expression. RTE-1 protein is another negative regulator of ET. ET induces the overexpression of the *RTE-1* gene and the product of this process induces a decrease plant sensitivity to ET [61]. Nevertheless, the influence of salinity stress is confirmed by the overexpression of the *WRK23* gene. The WRK23 protein confers tolerance to NaCl-stress in *A. thaliana* [62].

ET is derived from the amino acid methionine, which is converted to S-adenosylmethionine (SAM) by S-adenosylmethionine synthase. SAM is converted to 1-aminocyclopropane-1-carboxylic acid (ACC) ACC synthase (ACS) and then ACC is converted to CO_2_ and cyanide by ACC oxidase (ACO). In most plant species, ACS is encoded by multigene families, which are differentially regulated by various environmental and developmental factors. In tomato (*Solanum lycopersicum*, *syn. Lycopersicon esculentum*), nine *ACS* genes have been cloned and their expression studied. *LeACS6* transcripts normally accumulate in non-ripening fruit. The *Arabidopsis thaliana* genome encodes 12 *ACS*-like genes. *ACS3* is a pseudogene with a short sequence, whereas *ACS10* and *12* can complement the *Escherichia coli* aminotransferase mutant DL39 and are thus aminotransferases [63,64]. According to the TAIR database, the product of *ACS6* gene expression in *A. thaliana* tissues is involved in, among others, the ACC biosynthesis process, whereas the ACS10 protein does not have ACC synthase activity. Both *ACS6* and *ACS10* are expressed in leaves. The *ACO1* gene is expressed in *A. thaliana* leaves apex and the ACO1 enzyme plays a crucial role in ET biosynthesis. ACO1 and ACS6 proteins are located in the cytoplasm (https://www.arabidopsis.org/index.jsp (accessed on 29 December 2022)) [65]. No changes in the expression level of the *constitutive triple response 1 (CTR1)* gene were detected. CTR1 is another ET negative regulator, whose expression level is usually regulated in plants under salinity stress. In *ctr1* loss-of-function *Arabidopsis* mutants, higher salinity resistance was detected [66].

However, since ACC is a rate-limiting step in ET biosynthesis, ACS is considered a major target in determining the production of this phytohormone during stress response [31]. Most of the eight functional ACS genes found in *A. thaliana*, namely *ACS1*, *ACS2*, *ACS5*, *ACS6*, *ACS7*, and *ACS8*, as well as their respective homologs, were found to be upregulated during the salinity stress response in different plants, mostly glycophytes [67,68,69,70,71]. Moreover, Ellouzi et al. (2014) [72] reported that halophyte representatives, namely *Cakile maritima* and *Thellungiella salsuginea* responded to short-term salinity stress with intensive ACC accumulation, and somehow weaker ACC accumulation was described in the mentioned study for the glycophyte representative, namely *A. thaliana*. In all reported cases, ACC accumulation was assessed up to 72 h past salinity stress initiation. Thus, the mentioned studies unequivocally support the assumption that salinity stress induces the production of ACC, mainly by upregulated *ACC* gene expression. Here, we analysed the circadian rhythm of *ACS6* homolog expression in salinity-stressed common ice plants. Contrary to the mentioned experimental data, we found that 14-day-long salinity stress resulted in *ACS6* suppression in all time points of the analysed circadian rhythm. This result was, to some extent, supported by the ACC amount analysis, and we also found that 14-day long salinity stress presence had a minute effect on the circadian rhythm of ACC leaf concentration. Besides ACS, ACO represents the second key enzyme in the ET biosynthesis pathway. According to earlier studies, several ACO genes expression as well as protein activity were upregulated in response to salinity stress [70,73]. However, a different salinity stress response, precisely *ACO1* downregulation, was described for wheat [74]. In our semi-halophyte model plant, we found a completely different salinity stress response of *ACO* gene family members. Similar to the ACS genes family member described earlier, 14-day-long salinity stress downregulated *ACO1* expression in all time points of the analysed circadian rhythm. We believe that the insight presented here into the circadian rhythm of the expression of semi-halophyte *ACS* and *ACO* genes family members represents an image of the steady-state established past the 14-day-long salinity treatment. One can speculate that during the very first hours of salinity stress response, the circadian rhythm of the expression of an ice plant’s *ACS* and *ACO* orthologs, and resulting ACC concentration, could be completely different. Moreover, while experimental data suggest the precise control of ET biosynthesis, our results may simply indicate that at the analysed stage of stress response the common ice plant required no additional ET.

## 4. Materials and Methods

### 4.1. Plant Cultivation

*M. crystallinum* L. seeds from one set (from the collection of the Botanical Garden of the Technical University of Darmstadt, Germany) were sown onto soil substrate in a greenhouse under controlled conditions of light (250–300 μmol photons m^−2^ s^−1^ of photosynthetically active radiation (PhAR)), relative humidity (RH) (50–60%), and 16/8 h day/night period. The substrate implemented in the experiment was made of the market-available soil “Aro” and sand (grain size in the range of 1–2 mm) mixed in a 4:1 *v*/*v* ratio. Two weeks after sowing, each seedling with a fully developed second leaf pair was transferred to an individual 0.4 L pot with 360 ± 0.1 g of the mentioned substrate applied per pot. After 6 weeks, the plants were divided into two groups: the first group was irrigated with tap water (control), and the second group was irrigated with 0.4 M NaCl (NaCl-treated). After 14 days of treatment with 8-week-old plants, CAM development in the NaCl-treated plants was confirmed by the measurement of the diurnal Δ-malate, a hallmark of functional CAM photosynthesis expressed as the difference in cell sap malate concentration between the beginning and the end of the light phase. Δ-malate was measured according to the method previously described for Clusia hilariana Schltdl [75]. With the CAM presence confirmed, the fourth pair of leaves of the control and NaCl-treated plants were collected for transcriptome analysis (*n* = 8). To determine the diurnal regulation of ET biosynthesis in salinity stress-affected plants, for biochemical and molecular analysis, the mentioned leaf pairs of both control and NaCl-treated plants were collected every 6 h during a 24-h-long course (5 time points), immediately frozen in liquid nitrogen, ground, and then stored at −80 °C.

### 4.2. Reflectance Measurement

Reflectance parameters: normalized difference vegetation index (NDVI), photochemical reflectance index (PRI), and carotenoid reflectance index (CRI) measurements were performed on the fourth leaf pairs of control and NaCl-treated *M. crystallinum* L. plants (*n* = 4 for each type of plants) after 14 days from the start point of NaCl irrigation using handheld PolyPen RP410 (Photon Systems Instruments, Drasov, Czech Republic). 

### 4.3. Gas Exchange Parameters Measurement

The measurement of the gas exchange was taken with the use of a gas analyser (Li-6400, Li-Cor, Lincoln, NE, USA) equipped with an LED light source (6400-02). The stomatal conductance, CO_2_ assimilation, transpiration, and internal CO_2_ concentration measurements were taken under conditions of photosynthetically active radiation (PAR). The measurements were made six times for three replicates of control and NaCl-treated *M. crystallinum* L. plants after 14 days from the start point of NaCl irrigation (1 replicate = 1 leaf of the fourth pair, 18 measurements in total for each parameter) between 10.00 a.m. and 4.00 p.m. The average value of all replicates for each parameter was calculated. 

### 4.4. RNA Isolation

Total RNA was isolated from fine-powdered ice plant leaf tissues with an Aurum™ Total RNA Mini Kit (Bio-Rad, Hercules, CA, USA) according to the method previously described [39]. For the removal of DNA contamination, digestion with DNase I (DNA I Amplification Grade, Merck, Darmstadt, Germany) was used. Preliminary RNA purity and quantity were determined using a Biospec-Nano (Shimadzu, Japan). To assess the integrity and purity of the RNA, the extracted RNA was separated by electrophoresis on agarose (1.5%) gels stained with EtBr. The bands were visualised on a Molecular Imager^®^ ChemiDoc™ XRS+ Imaging System (Bio-Rad, Hercules, CA, USA). For the transcriptome analysis, the quality of each isolated RNA sample/replicate was assessed with the Agilent 2100 Bioanalyser (Agilent, Santa Clara, CA, USA).

### 4.5. RNA Sequencing

The single sequencing library was prepared by a random fragmentation of the cDNA sample, followed by 5′ and 3′ adapter ligation. Sixteen cDNA libraries were prepared using the TrueSeq stranded mRNA Library Kit (Illumina, San Diego, CA, USA). Adapter-ligated fragments were then PCR amplified and gel purified. For cluster regeneration, the library was loaded into a flow cell where fragments were captured on a lawn of surface-bound oligos complementary to the library adapters. High-throughput RNA sequencing in PE151 (paired ends mode, with 151 bp read length) was performed by the Macrogen (Amsterdam, The Netherlands) using an Illumina NovaSeq6000 (Illumina, San Diego, CA, USA). All RNA-seq datasets generated for this study were deposited in the NCBI SRA database under BioProject PRJNA1089077.

### 4.6. Bioinformatics Analysis

Raw reads in the FASTQ format were subjected to qualitative analysis using the FastQC tool v. 0.11.5 (https://www.bioinformatics.babraham.ac.uk/projects/fastqc/ (accessed on 15 October 2022)) [76]. The Trinity program package [77] according to the RNA-seq experiment analysis protocol published in Nature Protocols [78] was used to analyse data. In addition, the Trimmomatic program was implemented [79] to remove adapters and cut off poor-quality readings. The quality filter was as follows: a Phred score (Q) = 20, minimal read length = 25 bp, and unpaired reads were excluded. 

Next, cleaned reads were used for de novo assembly using the Trinity v2.4.0 (https://github.com/trinityrnaseq/trinityrnaseq/wiki (accessed on 20 July 2022)). The putative function of the assembled unigenes and transcripts was determined using the Trinotate ver. 3.1.0 (https://trinotate.github.io (accessed on 11 August 2022)) [80]. Protein coding regions for all transcripts were predicted using TransDecoder ver. 5.0.1 [78] and were identified using BLASTX and BLASTP. The identification of functional protein domains (HMMER/PFAM) was also carried out [81,82] and potential protein signals and transmembrane domains (SignalP/tmHMM) were predicted [83]. 

In the next step, the reads, for each sample separately, were mapped to a set of assembled transcripts using the Bowtie2 aligner [84]. The number of mapped reads to unigenes and transcripts for each library was calculated with RSEM [85] and normalisation was done by specifying the number of fragments per kilobase of exon per million mapped fragments (FPKM). Differentially expressed genes (DEGs) were calculated using EdgeR [86], Deseq2 [87], and Limma [88]. The p values were adjusted for multiple testing using the Benjamini–Hochberg method. A corrected p value of 0.05 and log2 fold-change of ±1 were set as the threshold for significant differential expression. The GO (gene ontology) enrichment of differentially expressed genes (DEGs) was carried out using the topGO package [89] from R ver. 3.6.3, and also the ClusterProfiler ver. 3.6.0 (http://www.bioconductor.org/packages/release/bioc/html/clusterProfiler.html (accessed on 10 November 2022)) [90].

### 4.7. qPCR

Reverse transcription was carried out on 1000 ng of total RNA with an iScript cDNA Synthesis Kit (Bio-Rad, Hercules, CA, USA). During qPCR, the samples were labelled with iQ™ SYBR^®^ Green Supermix (Bio-Rad, Hercules, CA, USA) fluorescent dye. For a single reaction, 10–20 ng of cDNA and 150 nM of gene-specific primers were used (Supplementary Table S3). Each reaction consisted of 40 cycles and was performed in 4 repetitions. To test the amplification specificity, a dissociation curve was acquired by heating samples from 60 °C to 95 °C. The ubiquitin gene (Acc. no AF053563.1) was used as a housekeeping reference gene. The reaction efficiency was tested by serial dilutions of cDNAs with gene-specific primers. The expression was calculated using at least three reactions with an unstressed control (C_3_) from the first time point as calibrators according to a previously described method [91].

### 4.8. HPLC-MS Analysis of ACC Content

ACC content in *M. crystallinum* plants was measured according to the method described by Müller and Munne-Bosch (2011) [92] with some modifications. About 500 mg of frozen leaf material, previously ground in liquid nitrogen, was extracted with 5 mL of extraction solvent (methanol:isopropanol, 20:80 (*v*/*v*) with 1% of glacial acetic acid) using ultrasonication (4–7 °C) for 30 min in a 15 mL Falcon tube. After centrifugation (10,000 rpm for 15 min at 4 °C), the supernatant was collected and the pellet was re-extracted with 1 mL of extraction solvent and this step was repeated three times. Then, supernatants were combined and dried completely under a nitrogen stream and re-dissolved in 500 μL of methanol and filtered through a 0.22 μm PTFE filter (Waters, Milford, MA, USA). Samples (5 μL) were then analysed by UPLC/ESI-MS/MS. The HPLC analysis was conducted using a Shimadzu LCMS-2020 (JPN) system with an autosampler. Plant extract separation was carried out using a Kinetex 2.6 µm C18 100 × 2.1 mm column. The flow rate of the eluent was maintained at 0.400 mL/min. The gradient profile was as outlined in Müller and Munne-Bosch (2011) [92]. MS analysis was conducted with a Shimadzu quadrupole mass spectrometer in positive ion mode for ACC analysis. The MS settings included the following: DL temperature at 250 °C, HB temperature at 200 °C, detector voltage at 0.95 kV, oven temperature at 35 °C, and a nebulizing gas flow of 15 L/min. ACC concentration in plant tissues was quantified using the external standard calibration curve method, preparing five standard solutions ranging from 0.05 to 10 ng/µL.

### 4.9. Statistical Analyses 

All statistical analyses of results were performed with Statistica 13 (Statsoft, Tulsa, OK, USA) software. For pairwise comparisons, the Student’s *t*-test was used. The data were subjected to a one-way analysis of variance (ANOVA).

## 5. Conclusions

The results collected in this report indicate that in semi-halophytes, the sustained stress of prolonged salinity stress does not have a significant impact on the main components of biosynthesis and molecular mechanisms involved in its regulation. It is possible that, at the analysed moment of plant–environment interaction, the involvement of such potent phytohormones and modifications implemented in ET production during the very first hours of the plant–environment interaction meets the current demand. The view emerging from the assessed results may represent the steady state that is established in semi-halophytes during prolonged salinity stress response. 

## Figures and Tables

**Table 1:** Genes related to ethylene (ET) (ERGs), abscisic acid (ABA) catabolic process, nitric oxide biosynthetic process, regulation of stomatal movement, reductive pentose-phosphate cycle, glycine catabolic process, chlorophyll binding, photosystem I, photosystem II, starch binding, and plant-type vacuole differentially expressed in *Mesembryanthemum crystallinum* L. leaves under NaCl treatment.

Gene Name	Annotated Species	Description	Unigene ID	log_2_ Fold Change NaCl-Treated vs. Control	*p* Value
**Ethylene (ET)-related genes (ERGs)**					
*ACO*	*A. deliciosa*	1-aminocyclopropane-1-carboxylate oxidase	TRINITY_DN78175	3.0	2 × 10^−24^
			TRINITY_DN69902	2.8	4 × 10^−19^
*ACO5*	*A. thaliana*	1-aminocyclopropane-1-carboxylate oxidase	TRINITY_DN31732	−1.5	5 × 10^−4^
*ETR*	*P. persica*	Ethylene receptor	TRINITY_DN29696	1.7	4 × 10^−4^
*RTE1*	*A. thaliana*	Protein reversion-to-ethylene sensitivity1	TRINITY_DN30452	2.2	1 × 10^−6^
*RAP2.12*	*A. thaliana*	Ethylene-responsive transcription factor RAP2-12	TRINITY_DN11475	1.8	1 × 10^−12^
			TRINITY_DN18663	2.4	3 × 10^−7^
*RAP2.3*	*A. thaliana*	Ethylene-responsive transcription factor RAP2-3	TRINITY_DN19854	1.5	2 × 10^−8^
*RAP2.4*	*A. thaliana*	Ethylene-responsive transcription factor RAP2-4	TRINITY_DN50762	1.3	4 × 10^−4^
*ERF53*	*A. thaliana*	Ethylene-responsive transcription factor ERF053	TRINITY_DN24801	−2.0	9 × 10^−10^
*ERF61*	*A. thaliana*	Ethylene-responsive transcription factor ERF061	TRINITY_DN79349	2.9	4 × 10^−12^
*ERF80*	*A. thaliana*	Ethylene-responsive transcription factor 9	TRINITY_DN32188	2.0	8 × 10^−4^
*AP2L1*	*A. thaliana*	AP2-like ethylene-responsive transcription factor At1g16060	TRINITY_DN25103	1.2	7 × 10^−5^
*RAV2*	*A. thaliana*	AP2/ERF and B3 domain-containing transcription repressor RAV2	TRINITY_DN25847	−4.4	7 × 10^−13^
*WRKY23*	*A. thaliana*	WRKY transcription factor 23	TRINITY_DN33449	1.4	4 × 10^−6^
**Abscisic acid (ABA) catabolic process (GO:0046345)**					
*PPCK1*	*A. thaliana*	Phosphoenolpyruvate carboxylase kinase 1	TRINITY_DN29388	4.5	7 × 10^−17^
			TRINITY_DN25641	3.7	1 × 10^−7^
*ATHB7*	*A. thaliana*	Homeobox-leucine zipper protein ATHB-7	TRINITY_DN28056	2.6	1 × 10^−17^
			TRINITY_DN23684	2.6	7 × 10^−15^
			TRINITY_DN89546	2.4	1 × 10^−17^
			TRINITY_DN29717	2.2	3 × 10^−26^
*BEL1*	*A. thaliana*	Homeobox protein BEL1 homolog	TRINITY_DN18515	2.1	2 × 10^−13^
*BELH1*	*A. thaliana*	BEL1-like homeodomain protein 1	TRINITY_DN27069	1.9	4 × 10^−21^
*CAR4*	*A. thaliana*	Protein C2-domain ABA-related 4	TRINITY_DN24769	3.7	3 × 10^−5^
			TRINITY_DN17184	2.7	4 × 10^−12^
*P2C24*	*A. thaliana*	Probable protein phosphatase 2C 24	TRINITY_DN25846	3.9	3 × 10^−20^
*P2C37*	*A. thaliana*	Protein phosphatase 2C 37	TRINITY_DN2812	1.2	5 × 10^−8^
*P2C56*	*A. thaliana*	Protein phosphatase 2C 56	TRINITY_DN30187	2.0	8 × 10^−11^
*ALFC2*	*P. sativum*	Fructose-bisphosphate aldolase 2	TRINITY_DN12822	2.9	2 × 10^−6^
*PIP22*	*A. thaliana*	Aquaporin PIP2-2	TRINITY_DN16293	2.4	1 × 10^−8^
*ERD7*	*A. thaliana*	Protein early-responsive to dehydration 7	TRINITY_DN11051	2.6	3 × 10^−8^
*AFP2*	*A. thaliana*	Ninja-family protein AFP2	TRINITY_DN37779	3.5	8 × 10^−6^
*AMO*	*A. thaliana*	Primary amine oxidase	TRINITY_DN32642	2.8	5 × 10^−4^
			TRINITY_DN33051	3.5	8 × 10^−13^
*SAPK2*	*O. sativa*	Serine/threonine-protein kinase SAPK2	TRINITY_DN39476	2.9	2 × 10^−12^
*SRK2I*	*A. thaliana*	Serine/threonine-protein kinase SRK2I	TRINITY_DN37625	1.2	2 × 10^−6^
*CIPK1*	*A. thaliana*	CBL-interacting serine/threonine-protein kinase 1	TRINITY_DN29316	1.2	2 × 10^−5^
*Y1141*	*A. thaliana*	G-type lectin S-receptor-like serine/threonine-protein kinase At1g11410	TRINITY_DN40172	1.6	2 × 10^−14^
*CRK*	*D. carota*	CDPK-related protein kinase	TRINITY_DN40076	1.6	2 × 10^−7^
*CDPKO*	*O. sativa*	Calcium-dependent protein kinase 24	TRINITY_DN32679	1.0	1 × 10^−14^
*RMR41*	*A. thaliana*	Remorin 4.1	TRINITY_DN21179	1.1	3 × 10^−5^
*AP2L1*	*A. thaliana*	AP2-like ethylene-responsive transcription factor At1g16060	TRINITY_DN25103	1.3	7 × 10^−5^
*CRPM4*	*A. thaliana*	Cold-regulated 413 plasma membrane protein 4	TRINITY_DN33710	1.7	7 × 10^−5^
*RGLG2*	*A. thaliana*	E3 ubiquitin-protein ligase RGLG2	TRINITY_DN33838	2.9	5 × 10^−5^
*GBLPA*	*A. thaliana*	Receptor for activated C kinase 1A	TRINITY_DN3696	1.8	2 × 10^−5^
*HHP1*	*A. thaliana*	Heptahelical transmembrane protein 1	TRINITY_DN40228	2.4	2 × 10^−13^
*ECP44*	*D. carota*	Phosphoprotein ECPP44	TRINITY_DN50318	1.8	1 × 10^−4^
*ANXD4*	*A. thaliana*	Annexin D4	TRINITY_DN59321	1.1	6 × 10^−5^
*GOLS2*	*A. thaliana*	Galactinol synthase 2	TRINITY_DN63076	3.6	4 × 10^−6^
*SRM1*	*A. thaliana*	Transcription factor SRM1	TRINITY_DN1724	1.3	6 × 10^−8^
*MYB88*	*A. thaliana*	Transcription factor MYB88	TRINITY_DN28071	1.3	1 × 10^−4^
*MYBS3*	*O. sativa*	Transcription factor MYBS3	TRINITY_DN9971	1.2	2 × 10^−5^
*MY102*	*A. thaliana*	Transcription factor MYB102	TRINITY_DN36820	1.1	5 × 10^−4^
*NAP2*	*S.lycopersicum*	NAC domain-containing protein 2	TRINITY_DN23251	1.6	1 × 10^−6^
*NAC2*	*A. thaliana*	NAC domain-containing protein 2	TRINITY_DN2528	1.5	8 × 10^−6^
*AL7B4*	*A. thaliana*	Aldehyde dehydrogenase family 7 member B4	TRINITY_DN49632	1.3	1 × 10^−9^
			TRINITY_DN23905	1.2	7 × 10^−4^
*CP29B*	*A. thaliana*	RNA-binding protein CP29B	TRINITY_DN42372	−1.6	9 × 10^−4^
**Abscisic acid catabolic process (GO:0046345), (+)-abscisic acid 8′-hydroxylase activity (GO:0010295)**					
*ABAH2*	*A. thaliana*	Abscisic acid 8′-hydroxylase 2	TRINITY_DN51717	−2.6	2 × 10^−17^
			TRINITY_DN36601	−2.3	9 × 10^−25^
*ABAH4*	*A. thaliana*	Abscisic acid 8′-hydroxylase 4	TRINITY_DN87577	−7.4	2 × 10^−18^
**Nitric oxide biosynthetic process (GO:0006809)**					
*NOS*	*A. thaliana*	Nitric oxide synthase	TRINITY_DN39531	1.8	1 × 10^−4^
*AMO*	*A. thaliana*	Primary amine oxidase	TRINITY_DN32642	2.8	5 × 10^−4^
			TRINITY_DN33051	3.5	8 × 10^−13^
**Regulation of stomatal movement (GO:0010119)**					
*BCA1*	*A. thaliana*	Beta carbonic anhydrase 1	TRINITY_DN1383	2.2	4 × 10^−15^
			TRINITY_DN16509	2.3	1 × 10^−7^
*BCA2*	*A. thaliana*	Beta carbonic anhydrase 2	TRINITY_DN16509	2.6	9 × 10^−5^
			TRINITY_DN1383	2.7	5 × 10^−28^
*CAH2*	*F. linearis*	Carbonic anhydrase 2	TRINITY_DN38373	1.6	1 × 10^−5^
*P2C37*	*A. thaliana*	Protein phosphatase 2C 37	TRINITY_DN2812	1.2	5 × 10^−8^
*P2C56*	*A. thaliana*	Protein phosphatase 2C 56	TRINITY_DN30187	2.0	8 × 10^−11^
*SRK2I*	*A. thaliana*	Serine/threonine-protein kinase SRK2I	TRINITY_DN39476	2.9	2 × 10^−12^
*ZIFL1*	*A. thaliana*	Protein zinc induced facilitator-like 1	TRINITY_DN54306	2.4	2 × 10^−12^
			TRINITY_DN38574	2.1	8 × 10^−12^
*AKT1*	*A. thaliana*	Potassium channel AKT1	TRINITY_DN28154	2.1	1 × 10^−5^
*NHX2*	*A. thaliana*	Sodium/hydrogen exchanger 2	TRINITY_DN68370	1.4	1 × 10^−20^
*MYB61*	*A. thaliana*	Transcription factor MYB61	TRINITY_DN31421	2.1	3 × 10^−8^
**Reductive pentose-phosphate cycle (GO:0019253), ribulose-bisphosphate carboxylase activity (GO:0016984)**					
*RBS1*	*M. crystallinum*	Ribulose bisphosphate carboxylase small chain 1	TRINITY_DN71513	−2.1	1 × 10^−9^
*RBS3*	*M. crystallinum*	Ribulose bisphosphate carboxylase small chain 3	TRINITY_DN28102	−1.2	2 × 10^−3^
			TRINITY_DN39730	−1.5	1 × 10^−3^
*RBS4*	*M. crystallinum*	Ribulose bisphosphate carboxylase small chain 4	TRINITY_DN24229	−3.2	5 × 10^−7^
*RBS5*	*M. crystallinum*	Ribulose bisphosphate carboxylase small chain 5	TRINITY_DN16432	−4.0	4 × 10^−17^
*RBS6*	*M. crystallinum*	Ribulose bisphosphate carboxylase small chain 6	TRINITY_DN90082	−1.9	9 × 10^−13^
*RAF2*	*A. thaliana*	Rubisco accumulation factor 1.2, chloroplastic	TRINITY_DN4605	−1.1	3 × 10^−20^
**Reductive pentose-phosphate cycle (GO:0019253)**					
*KPPR*	*M. crystallinum*	Phosphoribulokinase	TRINITY_DN40068	−1.6	6 × 10^−7^
			TRINITY_DN78943	−1.3	4 × 10^−7^
			TRINITY_DN42604	−1.2	3 × 10^−5^
*G3PB*	*A. thaliana*	Glyceraldehyde-3-phosphate dehydrogenase GAPB	TRINITY_DN43620	−1.7	1 × 10^−3^
*S17P*	*T. aestivum*	Sedoheptulose-1,7-bisphosphatase	TRINITY_DN88837	−1.2	2 × 10^−5^
**Glycine catabolic process (GO:0006546)**					
*GCSH*	*F. anomala*	Glycine cleavage system H protein, mitochondrial	TRINITY_DN54747	−2.1	4 × 10^−10^
	*M. crystallinum*		TRINITY_DN70038	−1.8	3 × 10^−12^
*GCST*	*M. crystallinum*	Aminomethyltransferase, mitochondrial	TRINITY_DN22073	−1.7	2 × 10^−6^
			TRINITY_DN78482	−1.3	4 × 10^−14^
*GCSP*	*S. tuberosum*	Glycine dehydrogenase (decarboxylating), mitochondrial	TRINITY_DN92007	−2.1	1 × 10^−6^
			TRINITY_DN95999	−1.9	5 × 10^−9^
**Chlorophyll binding (GO:0016168), chloroplast thylakoid membrane (GO:0009535), photosystem I (GO:0009522), photosystem II (GO:0009523), photosynthesis, light harvesting (GO:0009765)**					
*CB2A*	*S. oleracea*	Chlorophyll a-b binding protein	TRINITY_DN60950	−3.3	6 × 10^−4^
			TRINITY_DN38282	−3.0	2 × 10^−3^
			TRINITY_DN4800	−4.2	6 × 10^−5^
*CB2D*	*S. lycopersicum*	Chlorophyll a-b binding protein 1D	TRINITY_DN22522	−4.6	5 × 10^−12^
			TRINITY_DN30304	−2.5	1 × 10^−5^
			TRINITY_DN40637	−3.7	2 × 10^−4^
			TRINITY_DN68714	−4.1	2 × 10^−4^
			TRINITY_DN80965	−2.3	4 × 10^−4^
			TRINITY_DN30304	−2.8	2 × 10^−3^
			TRINITY_DN11494	−2.9	3 × 10^−3^
*CB4C*	*A. thaliana*	Chlorophyll a-b binding protein CP29.3	TRINITY_DN42750	−1.6	7 × 10^−4^
*CB21*	*S. latifolia*	Chlorophyll a-b binding protein	TRINITY_DN38282	−2.7	5 × 10^−24^
			TRINITY_DN51262	−2.6	6 × 10^−20^
	*R. sativus*	Chlorophyll a-b binding of LHCII type 1 protein	TRINITY_DN63494	−3.0	2 × 10^−5^
			TRINITY_DN69269	−1.8	2 × 10^−3^
*CB23*	*N. tabacum*	Chlorophyll a-b binding protein 36	TRINITY_DN59686	−2.9	3 × 10^−7^
			TRINITY_DN38282	−2.6	3 × 10^−19^
			TRINITY_DN88301	−2.4	2 × 10^−10^
*CB27*	*N. tabacum*	Chlorophyll a-b binding protein 7	TRINITY_DN27874	−3.0	2 × 10^−3^
			TRINITY_DN28870	−3.8	4 × 10^−6^
			TRINITY_DN30304	−3.1	9 × 10^−4^
**Photosystem II (GO:0009523), chloroplast thylakoid membrane (GO:0009535), photosynthesis, light harvesting (GO:0009765)**					
*CP29B*	*A. thaliana*	RNA-binding protein CP29B	TRINITY_DN90726	−3.1	2 × 10^−12^
			TRINITY_DN42372	−1.6	9 × 10^−4^
*PSBQ1*	*A. thaliana*	Oxygen-evolving enhancer protein 3-1	TRINITY_DN17369	−1.6	3 × 10^−6^
**Photosystem I reaction centre (GO:0009538), photosystem I (GO:0009522), chloroplast thylakoid membrane (GO:0009535)**					
*PSAD*	*S. oleracea*	Photosystem I reaction centre subunit II	TRINITY_DN59085	−1.1	6 × 10^−4^
*PSAH*	*O. sativa*	Photosystem I reaction centre subunit VI	TRINITY_DN14879	−1.6	4 × 10^−6^
	*S. oleracea*		TRINITY_DN53439	−1.4	6 × 10^−5^
**Chloroplast thylakoid membrane (GO:0009535)**					
*PTAC5*	*A. thaliana*	Protein disulphide isomerase pTAC5	TRINITY_DN27918	−1.3	1 × 10^−19^
*PTA16*	*A. thaliana*	Protein plastid transcriptionally active 16	TRINITY_DN37868	−1.3	8 × 10^−8^
*CHL*	*A. thaliana*	Chloroplastic lipocalin	TRINITY_DN16390	−1.1	3 × 10^−8^
*CG160*	*A. thaliana*	Protein conserved in the green lineage 160	TRINITY_DN24793	−2.2	3 × 10^−19^
*CAO*	*A. thaliana*	Chlorophyllide a oxygenase	TRINITY_DN21455	−1.7	6 × 10^−5^
			TRINITY_DN31549	−2.5	3 × 10^−4^
	*O. sativa*		TRINITY_DN84817	−2.5	3 × 10^−7^
*DNJA6*	*A. thaliana*	Chaperone protein dnaJ A6	TRINITY_DN32102	−1.1	2 × 10^−14^
*TL29*	*A. thaliana*	Thylakoid lumenal 29 kDa protein	TRINITY_DN35144	−1.7	3 × 10^−9^
*STR4*	*A. thaliana*	Rhodanese-like domain-containing protein 4	TRINITY_DN20986	−2.5	3 × 10^−4^
*STR9*	*A. thaliana*	Rhodanese-like domain-containing protein 9	TRINITY_DN59649	−2.5	3 × 10^−7^
*ABA2*	*S. oleracea*	Zeaxanthin epoxidase	TRINITY_DN87513	−1.8	2 × 10^−5^
*CUT1A*	*A. thaliana*	Protein curvature thylakoid 1A	TRINITY_DN32471	−1.3	6 × 10^−4^
*CRR3*	*A. thaliana*	Probable NAD(P)H dehydrogenase subunit CRR3	TRINITY_DN50182	−1.2	1 × 10^−8^
*NDF5*	*A. thaliana*	Protein NDH-dependent cyclic electron flow 5	TRINITY_DN61095	−2.1	4 × 10^−22^
*NDHK*	*E. globus*	NAD(P)H-quinone oxidoreductase	TRINITY_DN6819	−1.2	2 × 10^−13^
**Protochlorophyllide reductase activity (GO:0016630)**					
*PORA*	*A. thaliana*	Protochlorophyllide reductase A	TRINITY_DN13370	−2.7	4 × 10^−7^
	*C. sativus*	Protochlorophyllide reductase	TRINITY_DN26265	−1.7	7 × 10^−4^
			TRINITY_DN68683	−1.7	2 × 10^−12^
*PORB*	*H. vulgare*	Protochlorophyllide reductase B	TRINITY_DN40740	−1.7	4 × 10^−10^
**Starch binding (GO:2001070)**					
*DPE2*	*A. thaliana*	4-alpha-glucanotransferase DPE2	TRINITY_DN29808	−3.3	1 × 10^−7^
*DSP4*	*C. sativa*	Phosphoglucan phosphatase DSP4, amyloplastic	TRINITY_DN33943	−4.3	1 × 10^−20^
**Plant-type vacuole (GO:0000325)**					
*PTR2*	*A. thaliana*	Protein NRT1/ PTR family 8.3	TRINITY_DN38391	−2.0	2 × 10^−8^
*CAX3*	*A. thaliana*	Vacuolar cation/proton exchanger 3	TRINITY_DN38001	−1.1	9 × 10^−15^
			TRINITY_DN40895	−1.5	3 × 10^−11^
*TPC1*	*A. thaliana*	Two pore calcium channel protein 1	TRINITY_DN6810	−1.5	4 × 10^−6^
*TIP11*	*A. thaliana*	Aquaporin TIP1-1	TRINITY_DN35041	−1.3	5 × 10^−5^
*TIP21*	*A. thaliana*	Aquaporin TIP2-1	TRINITY_DN40936	−3.4	1 × 10^−14^
*CEP1*	*A. thaliana*	KDEL-tailed cysteine endopeptidase CEP1	TRINITY_DN11687	−2.2	3 × 10^−33^
			TRINITY_DN28528	−1.9	2 × 10^−7^
			TRINITY_DN29270	−2.0	3 × 10^−25^
*AA5GT*	*D. caryophyllus*	Cyanidin 3-O-glucoside 5-O-glucosyltransferase (acyl-glucose)	TRINITY_DN39241	−3.5	3 × 10^−9^
			TRINITY_DN39972	−2.4	1 × 10^−8^
*AB3C*	*A. thaliana*	ABC transporter C family member 3	TRINITY_DN40021	−1.1	7 × 10^−5^
*AB8C*	*A. thaliana*	ABC transporter C family member 8	TRINITY_DN40157	−5.1	6 × 10^−49^
*NRT25*	*A. thaliana*	High affinity nitrate transporter 2.5	TRINITY_DN77992	−2.1	1 × 10^−4^
*ALMTC*	*A. thaliana*	Aluminium-activated malate transporter 12	TRINITY_DN33933	−1.4	3 × 10^−21^
*ALMT2*	*A. thaliana*	Aluminium-activated malate transporter 2	TRINITY_DN21972	−1.4	4 × 10^−6^
*ERDL6*	*A. thaliana*	Sugar transporter ERD6-like 6	TRINITY_DN56467	−2.1	1 × 10^−3^
*NCL*	*A. thaliana*	Sodium/calcium exchanger NCL	TRINITY_DN63539	−3.3	1 × 10^−12^
*RNHX1*	*A. thaliana*	Putative ribonuclease H protein At1g65750	TRINITY_DN15507	−3.0	1 × 10^−10^
*CYSEP*	*V. mungo*	Vignain	TRINITY_DN33759	−1.9	2 × 10^−6^
*OCT3*	*A. thaliana*	Organic cation/carnitine transporter 3	TRINITY_DN35062	−3.9	8 × 10^−16^

## Data Availability

Data is contained within the article or Supplementary Material.

## Supplementary Materials

The supporting information can be downloaded at: https://www.mdpi.com/article/10.3390/ijms25094777/s1.
