# Supplementary material for: How the Ethylene Biosynthesis Pathway of Semi-Halophytes Is Modified with Prolonged Salinity Stress Occurrence?
Source: Int J Mol Sci. 2024 Apr 27;25(9):4777. doi: 10.3390/ijms25094777 (PMC11083548; doi:10.3390/ijms25094777)
Supplement: Supplementary file 1 [file ijms-25-04777-s001.zip › Supplementary Table S1-S3 and Figure S1-S2.pdf]

Supplementary Table S1-S3 and Figure S1-S2

Table S1. Sequencing data statistics of 16 cDNA libraries

Treatment	Biological replication	Clean read count	Total bases [Gbp]	GC (%)	AT (%)	Q20 (%)	Q30 (%)
**Control (C3)**	C3_3	38,985,944	5.9	46.95	53.05	97.55	93.38
	C3_4	41,659,356	6.3	46.87	53.13	97.32	92.82
	C3_5	41,659,356	6.3	46.87	53.13	97.32	92.82
	C3_6	41,205,070	6.2	46.16	53.84	97.54	93.20
	C3_7	41,803,842	6.3	46.50	53.50	97.41	93.08
	C3_8	42,102,086	6.3	46.34	53.66	97.44	93.12
	C3_9	41,579,458	6.3	46.43	53.57	97.44	93.13
	C3_10	40,969,220	6.2	46.39	53.61	97.40	93.01
	**Average**	**41,245,542**	**6.3**				
	**Total**	**329,964,332**	**49.9**				
**NaCl-treated (CAM)**	CAM_1	41,869,116	6.3	46.06	53.94	97.58	93.42
	CAM_2	41,315,346	6.3	46.30	53.70	97.46	93.11
	CAM_3	42,406,100	6.4	45.64	54.36	97.51	93.24
	CAM_4	42,465,562	6.4	45.85	54.15	97.35	92.90
	CAM_5	41,028,044	6.2	46.30	53.70	97.36	93.06
	CAM_6	40,935,048	6.2	46.14	53.86	97.25	92.87
	CAM_7	41,827,538	6.3	46.45	53.55	97.45	93.16
	CAM_8	41,075,786	6.2	46.04	53.96	97.44	93.14
	**Average**	**41,615,318**	**6.3**				
	**Total**	**332,922,540**	**50.3**				

Read count: total number of reads, total read bases: total number of bases sequenced, GC – the overall %GC of all bases in all sequences, AT – the overall %AT of all bases in all sequences, Q20(%): ratio of bases that have Phred quality score of over 20; Q30(%): ratio of bases that have Phred quality score of over 30. Phred quality score expresses the accuracy of each nucleotide.

Table S2. Numerical summary of *de novo* transcriptome assembly of M. crystallinum *L.*

	Transcripts	Unigenes
Total number	150,442	129,206
N50 (bp)	1,403	1,080
Average length (bp)	815	694
Median length (bp)	450	397

Table S3. List of qPCR primer sets used in this study

Target gene	Accession number	Forward Primer (5′ > 3′)	Reverse Primer (3′ > 5′)	Annealing teemperature [℃]	Product size [bp]
UBQ	AF053563.1	CGCACCTTGGCTGACTACA	AACAACCAGACCATGCAACA	56	94
ACS6	FX943325.1	GCAGCTTCGATTGTCATCTC	TTAAGTCCTTGCTCGAACGA	56	109
ACS10	FX909269.1	CACAAGGAACCAGGAGTCAC	GCTGGGTCAATATGAGTGGG	56	112
ACO1	AF375993.1	TGGGGACAAAGCACCAAACT	GAGCCCCTTGATTAGGTCGG	56	79

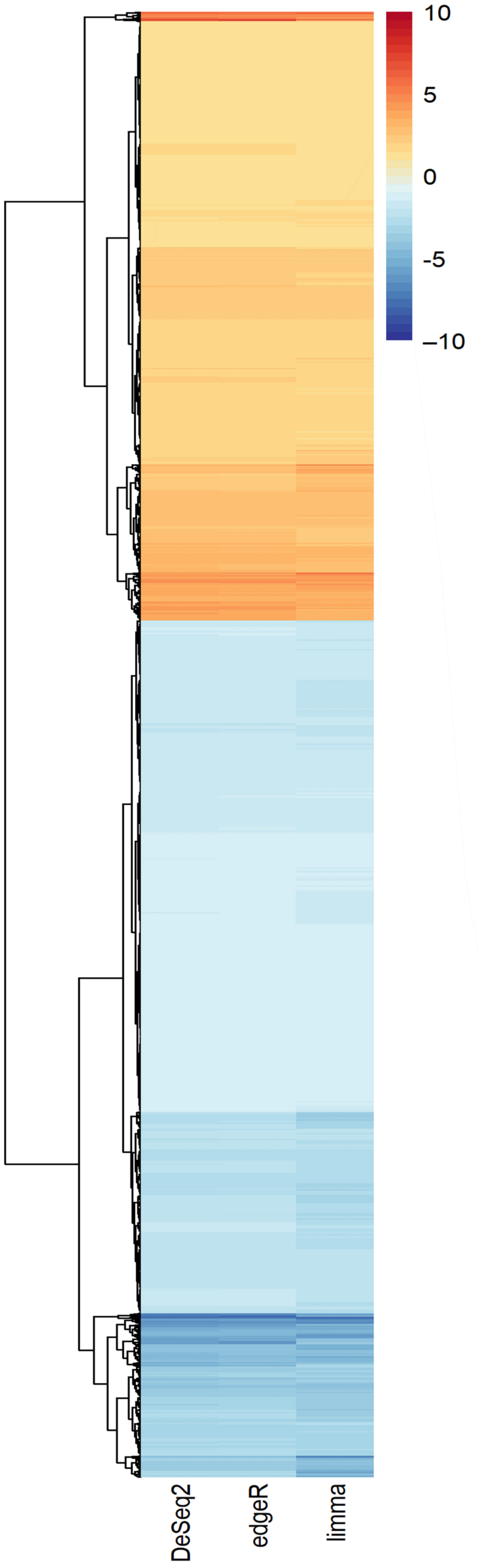

Figure S1. Heatmap showing the expression level of 3280 common genes identified using edgeR, DeSeq2 and limma tools; Up- and down-regulated DEGs were marked in orange and blue color, respectively.

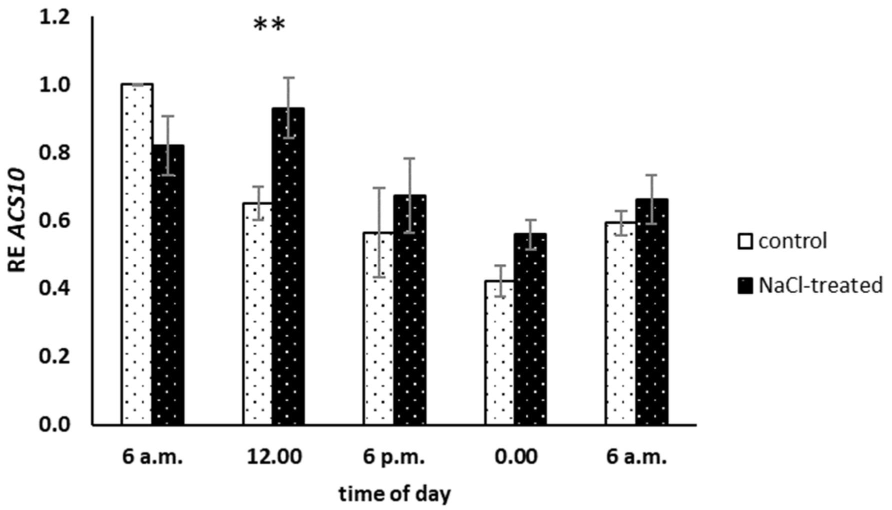

Figure S2. Expression of gene not involved in ethylene (ET) biosynthesis pathway, namely 1-aminocyclopropane-1-carboxylic acid (ACC) synthase 10 - *ACS10* analysed every 6 hours during 24-hours-long course in control and NaCl-treated *Mesembryanthemum crystallinum* L. plants. NaCl-treatment was applied for 14 days. Whiskers represent standard errors. The stars above indicate differences in expression between results obtained for C_3- and CAM (N=4) in one time point according to the t-Student test (*$p \leq 0.05$, **$p \leq 0.01$, ***$p \leq 0.005$).
